# Predictors of Functioning in Bipolar Disorder: Focused on Functioning Assessment Short Test (FAST)

**DOI:** 10.1192/bjo.2024.273

**Published:** 2024-08-01

**Authors:** Bo-Hyun Yoon, Hangoeunbi Kang

**Affiliations:** Naju National Hospital, Naju, Republic of Korea

## Abstract

**Aims:**

Functional outcome can be even more important than syndromic outcomes, as the ability to meet role expectations at work, home, or school and the quality of interpersonal relationships are often cited as the most important outcomes for people with bipolar disorder (BD) and their families. We investigated the factors correlated with functioning by using the Functioning Assessment Short Test (FAST).

**Methods:**

A total of 197 bipolar disorder out-patients were involved in this study, 166 (84.3%) were bipolar I disorder (BD-I) patients and 31 (15.7%) were bipolar II (BD-II) patients. We used the FAST for functioning of the patients and the severity of depressive and manic/hypomanic symptoms were measured by bipolar depression rating scale (BDRS) and Young Mania Rating Scale (YMRS). We also examined the disturbances in biological rhythm by the Biological Rhythm Interview of Assessment in Neuropsychiatry (BRIAN).

**Results:**

There were significant positive correlations between FAST and numbers of depressive episode, YMRS, BDRS and BRIAN and showed negative correlation between FAST and age at onset of mood disorder. FAST was associated with YMRS (β=0.3768, p < 0.001), BDRS (β=0.293, p < 0.001), BRIAN (β=0.167, p = 0.011), with 47.1% of the variance explained (R^2^=0.471, Durbin-Watson test = 1.51, p < 0.001) in multiple linear regression. In other words, residual mood symptoms and biological rhythm imbalance have a negative impact on the functioning of BD patients.

**Conclusion:**

Although the other factors must be present to predict the functioning of bipolar disorder patients, manic symptoms, depressive symptoms and biological rhythm imbalance have negative impacts on functioning of BD patients in this study.

